# Diversity oriented Deep Reinforcement Learning for targeted molecule generation

**DOI:** 10.1186/s13321-021-00498-z

**Published:** 2021-03-09

**Authors:** Tiago Pereira, Maryam Abbasi, Bernardete Ribeiro, Joel P. Arrais

**Affiliations:** Department of Informatics Engineering, Centre for Informatics and Systems of the University of Coimbra, University of Coimbra, Pinhal de Marrocos, Coimbra, Portugal

**Keywords:** Drug Design, SMILES, Reinforcement Learning, RNN

## Abstract

In this work, we explore the potential of deep learning to streamline the process of identifying new potential drugs through the computational generation of molecules with interesting biological properties. Two deep neural networks compose our targeted generation framework: *the Generator*, which is trained to learn the building rules of valid molecules employing SMILES strings notation, and *the Predictor* which evaluates the newly generated compounds by predicting their affinity for the desired target. Then, the Generator is optimized through Reinforcement Learning to produce molecules with bespoken properties. The innovation of this approach is the exploratory strategy applied during the reinforcement training process that seeks to add novelty to the generated compounds. This training strategy employs two Generators interchangeably to sample new SMILES: the initially trained model that will remain fixed and a copy of the previous one that will be updated during the training to uncover the most promising molecules. The evolution of the reward assigned by the Predictor determines how often each one is employed to select the next token of the molecule. This strategy establishes a compromise between the need to acquire more information about the chemical space and the need to sample new molecules, with the experience gained so far. To demonstrate the effectiveness of the method, the *Generator* is trained to design molecules with an optimized coefficient of partition and also high inhibitory power against the Adenosine $A_{2A}$ and $\kappa$ opioid receptors. The results reveal that the model can effectively adjust the newly generated molecules towards the wanted direction. More importantly, it was possible to find promising sets of unique and diverse molecules, which was the main purpose of the newly implemented strategy.

## Introduction

Drug development is a technically and financially complicated task which comprises several challenging stages until reaching the final objective [1]. The success rate in this task is relatively low, which makes some long-term research projects came to a fruitless end. As new diseases emerge or new treatment methods for the existing conditions are explored, it is evident that there is a need for an efficient and reliable drug development pipeline. Several computational strategies have been used to make this process more efficient and less likely to fail. In some techniques, virtual screening is used to reduce the search space. This technique reduces the chemical libraries search space by filtering the set of molecules according to desired properties [2]. However, this approach relies on the size and diversity of the initial set of molecules [3]. Contrarily, there are computational techniques for the de novo drug design that involves the exploration of the chemical space for the generation of new compounds from scratch, in an automated way [4]. Initially, the most successful algorithms included atom-based elongation or fragment-based combination and often joined with Evolutionary Algorithms (EA), or other global optimization techniques [5, 6].

Recent developments in Deep Learning (DL) have broadened the area of de novo molecule generation. As a result, it became a problem of inverse design in which the desirable properties are previously defined. Then, through Reinforcement Learning (RL) or other optimization methods, the chemical space that satisfies those properties is explored. Since then, these techniques have been successfully applied to hit discovery [7]. In this regard, Nicolaou et al. designed new molecules combining evolutionary techniques with graph-theory to perform a global search for promising molecules [8]. The findings obtained demonstrated the applicability of these methods, and its usefulness for in silico molecular design. More recently, several RL-based methods have also been widely employed in this area. Benjamin et al. in ORGANIC explored the combination of a Generative Adversarial Network (GAN) with RL to perform a biased molecular generation with specific attributes [9]. Other variants of RL methods, such as the REINFORCE, also lately applied in de novo drug design with encouraging results, showing that deep generative networks effectively model the Simplified Molecular Input Line Entry Specification (SMILES) representation of molecules using Recurrent Neural Networks (RNNs). Olivecrona et al. in REINVENT combined RNNs and RL to generate targeted molecules in the form of SMILES by learning an augmented episodic likelihood composed by a prior probability and a user-defined scoring function [10]. Moreover, Popova et al. implemented a model consisting of an RNN with stack-augmented memory as a computational generator and a Quantitative Structure-Activity Relationship (QSAR) model to estimate the molecular properties to be optimized by RL [11]. Other RL methods such as Deep Q-Learning are also proven to be a successful way of research. Zhou et al. designed new molecules with specific desired properties, formalizing the problem as a Markov Decision Process (MDP) and using a value function to solve [12].

Nevertheless, the computational generation of the lead compounds must always include specific fundamental properties. These molecular generative models must produce candidate molecules that are biologically active against the desired target and safe for the organism [13]. Moreover, it is essential to guarantee the chemical *validity* of the generated molecules, as well as their chemical and physical *diversity* [7, 14]. Although, the previous studies optimize specific single molecular properties and generate chemically valid molecules. However, diversity is often neglected in this topic [15]. Note that novelty is essential for drug candidate molecules since it is only by fulfilling this prerequisite is possible to discover alternative therapeutic solutions better than the existing ones [13, 16, 17]. In this regard, Liu et al. in DrugEx proposed a generative model to generate new active molecules against the Adenosine $A_{2A}$ receptors ($A_{2A}R$). DrugEx combines RNNs and RL with an exploration strategy to ensure greater chemical diversity in the obtained compounds [17]. The authors try to increase the diversity of molecules through the alternated use of two computational generators: one initially trained remains fixed and the other updated at each iteration through RL. However, this work suffers from the inefficient coverage of the chemical space when exploring new putative potential drugs. The second issue to overcome is the inability of the previous models to maintain the proportion of the valid molecules after the optimization for the biological property of interest [11]. In this work, we address these problems.

This paper proposes an end to end RL framework structured with RNNs, SMILES notation, and a policy gradient REINFORCE algorithm [18]. It is designed to generate lead compounds concerning their biological affinity for interesting receptors such as $A_{2A}R$ [19], and $\kappa -$opioid receptors (KOR) [20] or their partition coefficient. The goal is to obtain compounds with the optimized property and containing as much diversity as possible. Therefore, it proposes a new strategy to balance the exploration/exploitation dilemma, based on the two Generators methodology established in Liu et al. but with a valuable distinctive. The two generators in this scenario share the same architecture but with different internal parameters: one is more involved with exploration while the other is more focused on exploitation. The selection of each Generator to predict the next token is based on the previous evolution of the numerical reward. In addition, to prevent the repetitive generation of molecules, it creates a memory cell to be updated with the last generated molecules. There is a penalty for the reward whenever this set of molecules’ diversity decreased during exploring chemical space. Notwithstanding, besides the computational Generator, this framework is composed of a QSAR model for predicting the newly generated molecules’ affinity against the desired target. During the development of the QSAR, different architectures and molecular descriptors have been examined to obtain a robust model. Even though this work is directed at specific targets, it can be easily adapted to different biological interests goals.

## Methods

**Fig. 1:**
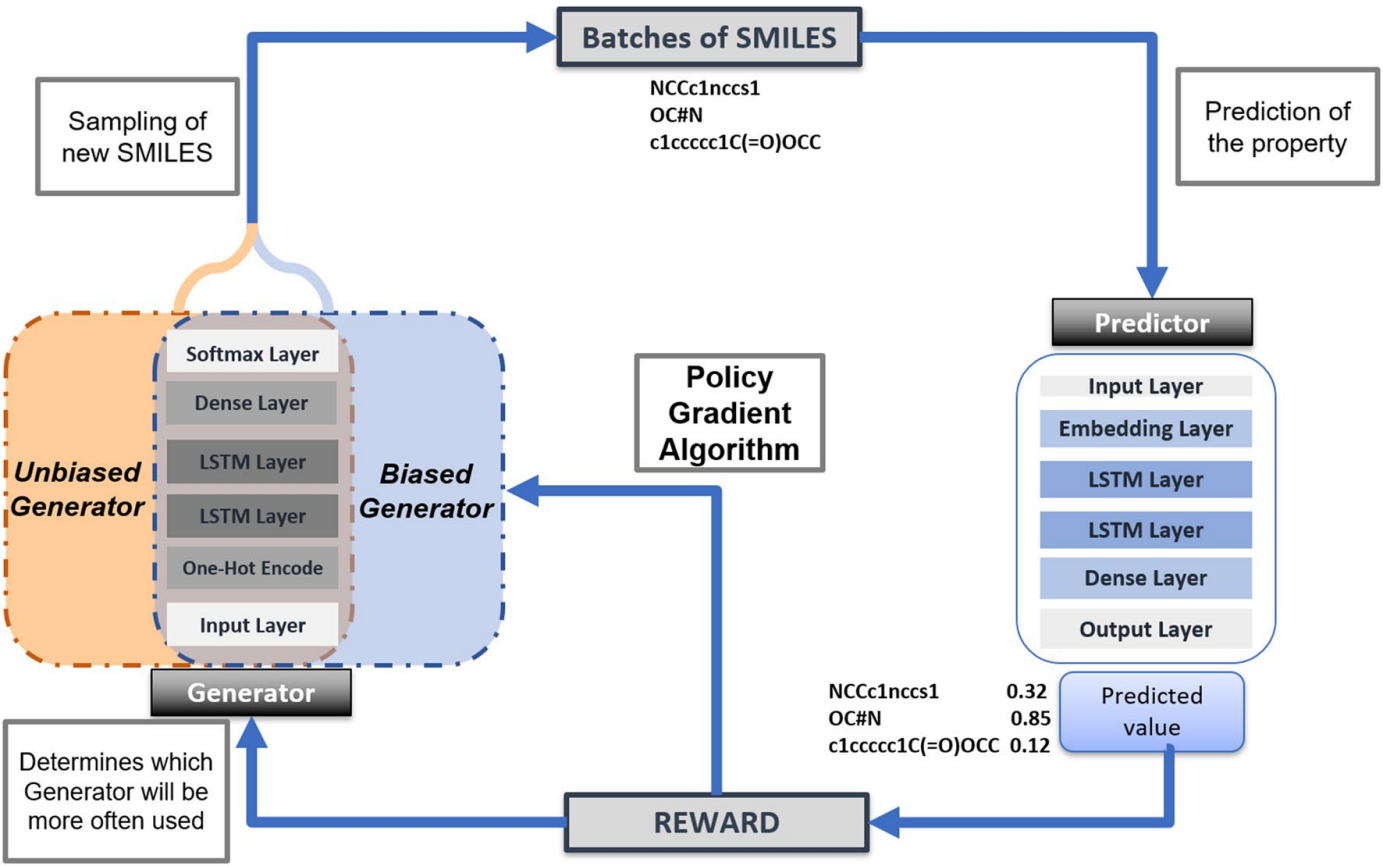
General overview of the framework: two Generators sharing the same architecture and the Predictor interconnected by Reinforcement Learning

The proposed framework is divided into two main parts. First, two deep neural networks are implemented using supervised learning: the generative and the QSAR models. The former is trained to learn the building rules of molecules using SMILES notation, and, the latter is trained to predict specific biological activities of compounds. Both are built with recurrent models, Long Short-Term Memory (LSTM) and Gated Recurrent Unit (GRU) cells, respectively, and using SMILES strings as input data. In the second step, the Generator will be re-trained through RL, and the Predictor will guide the training process to guarantee the generation of molecules with the optimized desired property. In this work, we are introducing a new strategy for the selection of tokens in the generation of SMILES strings and an approach to enhance novelty in the compounds. Therefore, the goal is to generate valid molecules with promising properties and, at the same time, to ensure a satisfactory diversity after application of RL to bias the Generator towards the desired purpose. Figure 1 describes the general workflow of the framework to perform a targeted lead generation.

### The generator model

The input data for this model are SMILES strings. Hence, it is necessary to perform some encoding to transform each sequential or structural character into a numerical value, capable of being used by the model. Data pre-processing starts by doing its tokenization, followed by the padding and, finally, by transforming it to one-hot encoding vectors. Tokenization involves the conversion of each atom or bond character into a char type (token). The vocabulary used in the construction of SMILES strings contained 45 tokens. Then, to standardize all the strings, starting and ending characters were added. The padding of the sequences ensures that all SMILES strings have 65 tokens. In this case, the starting character is ‘G’, the ending is ‘E’, and the padding is the space character. Finally, the SMILES strings are transformed into a one-hot encoding vector.

The architecture is similar to the one seen in the work of Gupta et al. [21]. It included an input layer, two LSTM layers with 256 units and 0.3 for the dropout value, applied between each layer. The dropout application can be seen as a regularization strategy that helps to minimize the learning inter-dependency and enhance the generalization of the model. Also, it has a densely connected layer with 43 units and a neuron unit with a Softmax activation function. Data was divided into batches of 16 SMILES, during 25 training iterations and the optimizer employed to update the weights was Adam with a learning rate of 0.001.

Throughout the training process, at each step, each token array is given as an input to the architecture, and the respective output is the probability distribution of all possible tokens. After several steps, the model is aimed to assign the highest probability to the real token, taking into account the rest of the sequence given as input. On that account, the “Teacher Forcing” algorithm is used during training. It means that the correct token is always integrated into the remaining set of tokens (to be used as input in the next step) and not the token derived from the model prediction, which minimizes the maximum-likelihood loss at each training step [22]. Consider $y = \{y_{1},y_{2},\ldots ,y_{T}\}$ as the correct output sequence for a given input sequence *X*, and ${\hat{y}}$ is the Generator output vector. Suppose we have *T* samples with each sample indexed by $t = 1,\ldots ,T$. The goal of training is to minimize the following cross-entropy loss function:

1 $$\begin{aligned} J(\theta ) = - \frac{1}{T} \sum _{t=1}^{T}\left[ y_t \log {\hat{y}}_{t} + (1-y_t) \log (1-{\hat{y}}_{t}) \right] \end{aligned}$$

**Fig. 2:**
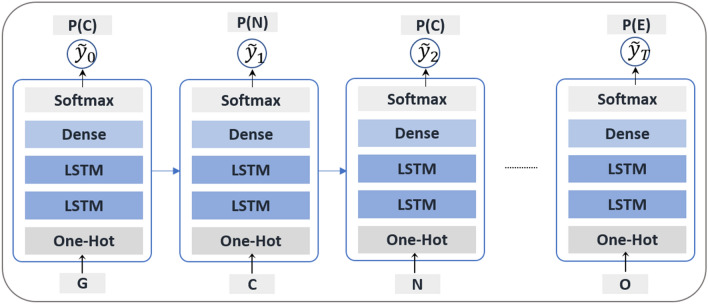
The outline of the training procedure for the SMILES string ‘GCNC(C)=OE’. For each step *t*, the input of the model is the vectorized token *y*_*t*_, and the output is the probability of the token predicted by the model being the correct token. The goal of the training process is to maximize this probability

The loss function (Eq. 1) is the negative log-likelihood ratio between the correct and competing tokens to guarantee that, after the training step, the output token is more likely to be chosen in future generations steps. The gradients are computed according to the clipping gradient method to avoid instability during training [23]. The combination of RNNs and sequential data such as SMILES notation has brought successful results in several fields, including in the generation of syntactically valid molecules [11]. This capability to learn the rules and dependencies inherent in the process of building molecules is explained by the ability of this type of architecture to learn essential input sections and retain them in their long-term memory to be used as appropriate [7]. Figure 2 shows the simplied depiction of the training procedure.

The last step is the output generation in which the new molecules are formed by predicting their constitution token by token. Therefore, at each generation step, the new atom or bond of the molecule is predicted, taking into account the structure already generated. Finally, the RDKit molecule sanitizer syntactically validates the newly generated molecules (http://www.rdkit.org).

### The predictor model

The Predictor is a QSAR model that performs the mapping between the structure of the molecules and its binding affinity against the targets of interest. Throughout this work, a compound’s binding affinity towards a target is evaluated using the parameter $\text{ IC}_{50}$. This parameter stands for the half-maximal inhibitory concentration, and it indicates how much of a substance is needed to inhibit $50\%$ of a given receptor. The value $p\text{ IC}_{50}$ is equal to $-\log (\text{ IC}_{50}$) since the nature of potency values is logarithmic. The higher values of $p\text{ IC}_{50}$ indicate exponentially more powerful inhibitors. Two distinct approaches were tested to determine the best architecture and molecular descriptor of the Predictor.

The first approach, used as the baseline, employs the Extended Connectivity Fingerprint (ECFP) as molecular representation. These bit vectors are widely used in the prediction of physicochemical properties, biological activity or toxicity of chemical compounds [24]. The model output is a real number, which is the estimated $\text{ pIC}_{50}$. The four developed algorithms are Support Vector Regression (SVR), Random Forest (RF), K-Neighrest Neighbors (KNN) and a deep Fully Connected Neural Network (FCNN). The input data was ECFP6 (vectors with 4096 bits), calculated with the RDkit Morgan Fingerprint algorithm with a three bonds radius [25]. The first three models have been implemented with the Scikit-learning tool (https://scikit-learn.org/). The parameters and hyperparameters applied in these ML-based QSARs are described in Table 1.

**Table 1:** Optimal hyperparameters for the standard QSAR models

SVR			RF		K-NN	
Kernel	C	Gamma	$\hbox {NumEstimators}^{[1]}$	$\hbox {MaxFeatures}^{[2]}$	K	Metric
’poly’	0.125	8	500	’sqrt’	11	Euclidean

${}^{[1]}$Number of decision trees in the forest.

${}^{[2]}$Maximum number of features considered for splitting a node. In this case, MaxFeatures = sqrt(n_features)

**Fig. 3:**
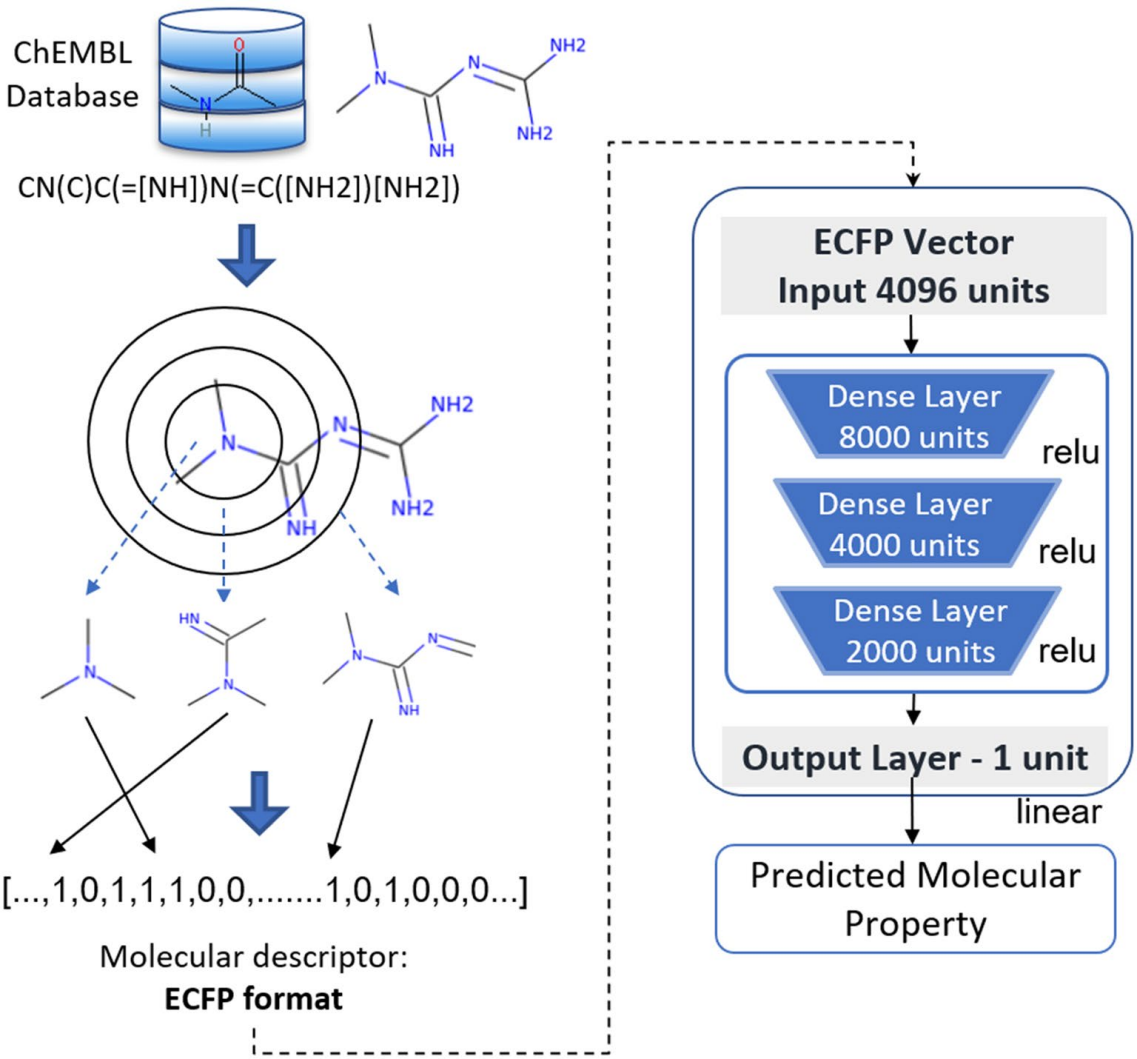
General schema of Predictor with FCNN architecture. ECFP vector is employed as input, calculated with the Morgan Fingerprint algorithm with a three bonds radius

The FCNN was implemented with three fully connected hidden layers (with 8000, 4000, and 2000 neurons) activated by Rectified Linear Unit (ReLU) with dropout technique between each fully connected layer to reduce the overfitting. The architecture includes an output layer, which consists of a single neuron that returns the estimation of the biological activity. Figure 3 depicts the model details.

**Fig. 4:**
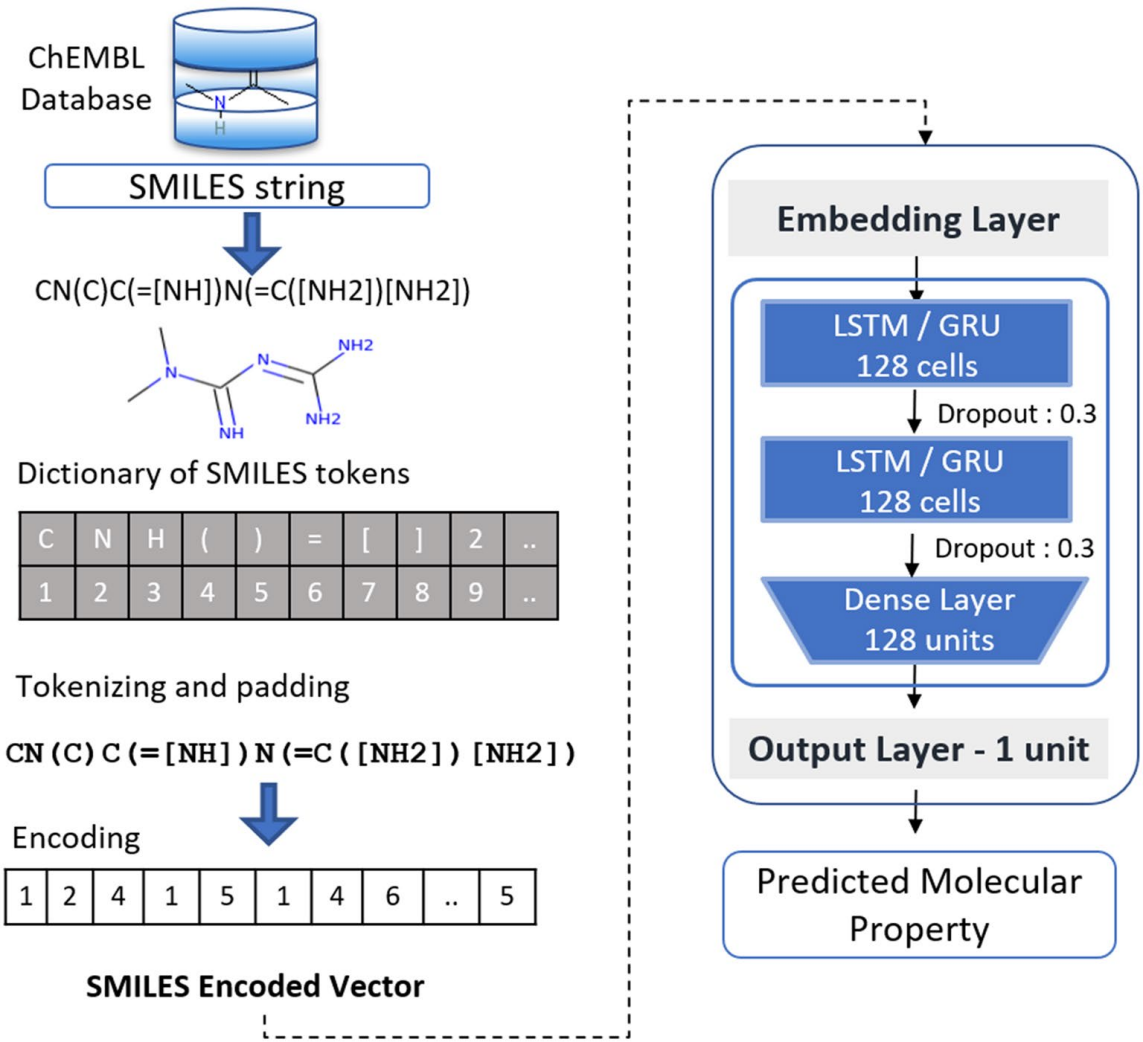
General schema of RNN-based Predictor architecture. SMILES encoding is transformed into an integer vector to be used as input

The second approach is depicted in Fig. 4, and it uses the SMILES strings as input data without converting them into any other form of descriptors. The model architecture consisted of the embedding layer that converts each token into a vector of 128 elements, two GRU layers (128 units), one dense layer activated by a ReLU function (128 units) and, finally, the output layer with a linear activation function.

The implementation starts with the pre-processing of SMILES in order to transform them into numeric descriptors. As with Generator, the main steps are tokenization and padding for each sequence. A dictionary is defined with the vocabulary that constitutes SMILES in terms of tokens. Then, through this dictionary, each token is mapped to a corresponding integer. This representation preserves the structural information, character and order, and has a low computational cost given the number of different tokens. Besides, it overcomes the issue of using indirect representations of chemical structures, which therefore adds human bias and in some cases, it can misrepresent the relationship between the compounds and the desired property [26].

Regarding architecture, in this case, instead of one-hot encoding, we apply an embedding layer. Thus, each integer representing a token is transformed into a dense vector that represents the projection of the token into a continuous vector space. As we used 128 units in the GRU layer, each token was also transformed into a vector with 128 elements. This notation allows to minimize the sparsity associated with one-hot encoding and allows the rest of the architecture to more easily assess the similarities and differences between the molecules so that the mapping between SMILES and biological activity can be more accurate.

The objective is to use the DL and recurrent architectures capabilities to identify the properties of the molecules responsible for causing interaction or not with the target of interest. After transforming the tokens into vectors of continuous values, they are fed to the two layers of GRU cells that will output a vector of 128 units. This vector contains relevant information extracted from SMILES and will be passed to a densely connected layer to transform the 128 unit vector into a single value. Finally, after applying a linear activation to this value, we obtain the predicted biological activity.

This type of architecture is particularly advantageous in QSAR modelling. First, due to the ability of GRU cells to hold a memory for the entire sequence, it is possible to identify valuable features throughout the molecule. Second, this architecture allows work with training data that have inputs of varying lengths. In opposition, traditional QSARs have a descriptor matrix with a fixed number of columns and the column position of every descriptor should remain fixed.

After determining the best parameters using a grid-search strategy, the implementation of the regression was performed using five-fold cross-validation to split the data and avoid unwanted overfitting. The data is divided into $85\%$ for training/validation and $15\%$ for testing. Then, the training/validation SMILES are divided into five folds to train an equal number of models. On each fold, data is randomly divided into $85\%$ of the training data $15\%$ for validation. The test set evaluates the robustness of the model in predicting the new molecule binding affinity. The loss function in this regression-like problem is the mean squared error. It helps to measure how close the Predictor learns to get the actual result. Moreover, the early stopping method is employed to allow specifying the arbitrarily large number of training epochs and stop training once the model performance stops improving on a validation subset. An important aspect that should be mentioned is the labels standardization of the data that the QSAR model will predict.

### Reinforcement learning strategy

The RL framework is implemented using the REINFORCE algorithm, and the aim is to teach the Generator the chemical spaces that guarantee the generation of molecules with bespoken properties. This learning process can be seen as an experience-driven change in behaviour. When a specific action brings us benefits, we learn to repeat it. This is the basis of RL, i.e., an agent that learns how to map states into actions through the maximization of the reward while interacting with the environment [27].

In other words, the goal of this type of problem is accomplished when the best policy/behaviour is progressively achieved, which is reflected in the maximization of the accumulated discounted reward from various actions [27]. In general, this formulation can be described using Eq. 2. On that account, a lower reward leads to incorrect behaviour/policy, whereas more substantial reward means that the behaviour/policy is evolving in the right direction.

2 $$\begin{aligned} R_{t} = \sum _{k=0}^{T} \gamma ^{k}r_{t+k+1} \end{aligned}$$

where $R_t$ is the return, t is the time step, T is the final time step, and $\gamma$ is a discount factor. It determines how much the future reward worths in the present [27]. Thus, it is a parameter varying from $0 \le \gamma < 1$ that makes the rewards in near time more desirable than those in the distant future.

The RL is based on the formal framework of the Markov decision problems (MDP). In this formalism, the agent interacts with its exterior, the environment, in order to select the best action depending on the state of the environment. At each step, the agent is in some state $s \in S$, and, it is necessary to choose an available action. In the next step, a numerical reward is assigned to this choice, evaluating the consequences of the previously taken action. In addition, the environment is updated, and the new state is presented to the agent to repeat the process [28]. The process includes the idea of cause and effect, a sense of non-determinism, and the existence of explicit goals.

This formalism can be adapted for the generation of molecules with SMILES strings, and we will specify the parallels between classical formalism and the deep generative field. Thus, the set of actions that the agent can choose corresponds to all characters and symbols that are used in the construction of valid SMILES. The states through which the agent can pass corresponds to the set of all SMILES strings that can be constructed during the generation process. The policy ($\pi$) maps the current state to the distribution of probabilities for choosing the next action [28]. Thus, the main objective of RL is to find an optimal policy, i.e., a policy that selects the actions to maximize the expected reward. The policy is the cornerstone of RL, and, in this case, it corresponds to *the Generator*. The weights of the Generator will be updated based on the gradient of a scalar performance measure ($J( \theta )$ in Eq. 3) with respect to the policy parameters. The aim is to maximize this performance objective so that their updates approximate gradient ascent in *J*:

3 $$\begin{aligned} \theta _{t+1} = \theta _{t} + \alpha \nabla J(\theta _t), \end{aligned}$$

where *t* represents the time step, $\theta$ the policy parameters, $\alpha$ the learning rate, and $\nabla J(\theta _{t})$ is an estimate, through its expectation, of the gradient of the performance measure with respect to $\theta _t$ [27]. Equation 4 represents the REINFORCE update which is achieved by using this sample to instantiate our generic stochastic gradient ascent algorithm (Eq. 3).

4 $$\begin{aligned} \theta _{t+1} = \theta _{t} + \alpha \gamma ^{t} R_{t} \nabla ln \pi (A_{t}|S_{t},\theta _{t}) \end{aligned}$$

### Learning process steps

The learning process will begin with the sampling of the new compounds. At this point, batches of SMILES are generated, and the analysis of each action is done token by token. In other words, the molecule is created and is assigned with a reward from the Predictor. Then, since each sampled token corresponds to an action, the molecule is “decomposed” into each of its tokens and “reconstructed” again to analyze the probability of each action being taken to calculate the loss function. The cumulative loss is the result of the sum of the probabilities analysis from each taken action in each molecule of the generated batch. On that account, the formulation approximates an MDP since, at each step of the molecule reconstruction, an action is chosen. In response to this action, a discounted format of the initial reward is assigned, and a new state is presented to the agent. This state corresponds to the junction of the partially reconstructed molecule with the selected token from the previous step. The newly created state is used to predict the next token/action.

The reward is not attributed to each action but assigned to each molecule. The better the prediction according to the objective, the greater will be the reward. After following this procedure for the batch of molecules, the loss function is calculated and, following the gradient descent method, the weights of the Generator are updated using the following loss function:

5 $$\begin{aligned} J(\theta _t) = -\frac{1}{n} \sum _{i=1}^{|S|} \sum _{j = 1} ^{ \text{ length }(s_i)} R_i \cdot \gamma ^i \cdot \ln {(p(s_j|s_0 \ldots s_{j-1},\theta )} ), \end{aligned}$$

As we are applying RL, the exploration/exploitation dilemma has to be considered to guarantee a reliable model. The exploration seeks to collect more information about the environment to select better actions in the future, while the exploitation favours the maximization of immediate rewards. As the agent perceives what gives him more and less reward, it is possible to decide if it is more important to continue learning or to pursue the most promising strategy with the experience gained so far [27]. We consider three strategies to address this issue throughout the work.

First, to make the sampling of new SMILES strings, we use a Softmax activation with a temperature parameter. The temperature allows to precisely control the randomness of predictions by scaling the logits before applying Softmax [21]. If the temperature is reduced, the actions that are already more likely to be chosen are favoured, making the model less susceptible to discovering new actions and, as such, it favours more the exploitation. On the other hand, using higher temperatures, the probabilities of each action tend to get closer, so it is possible to select different actions, and, as such, it is a strategy that gives more priority to the exploration.

**Fig. 5:**
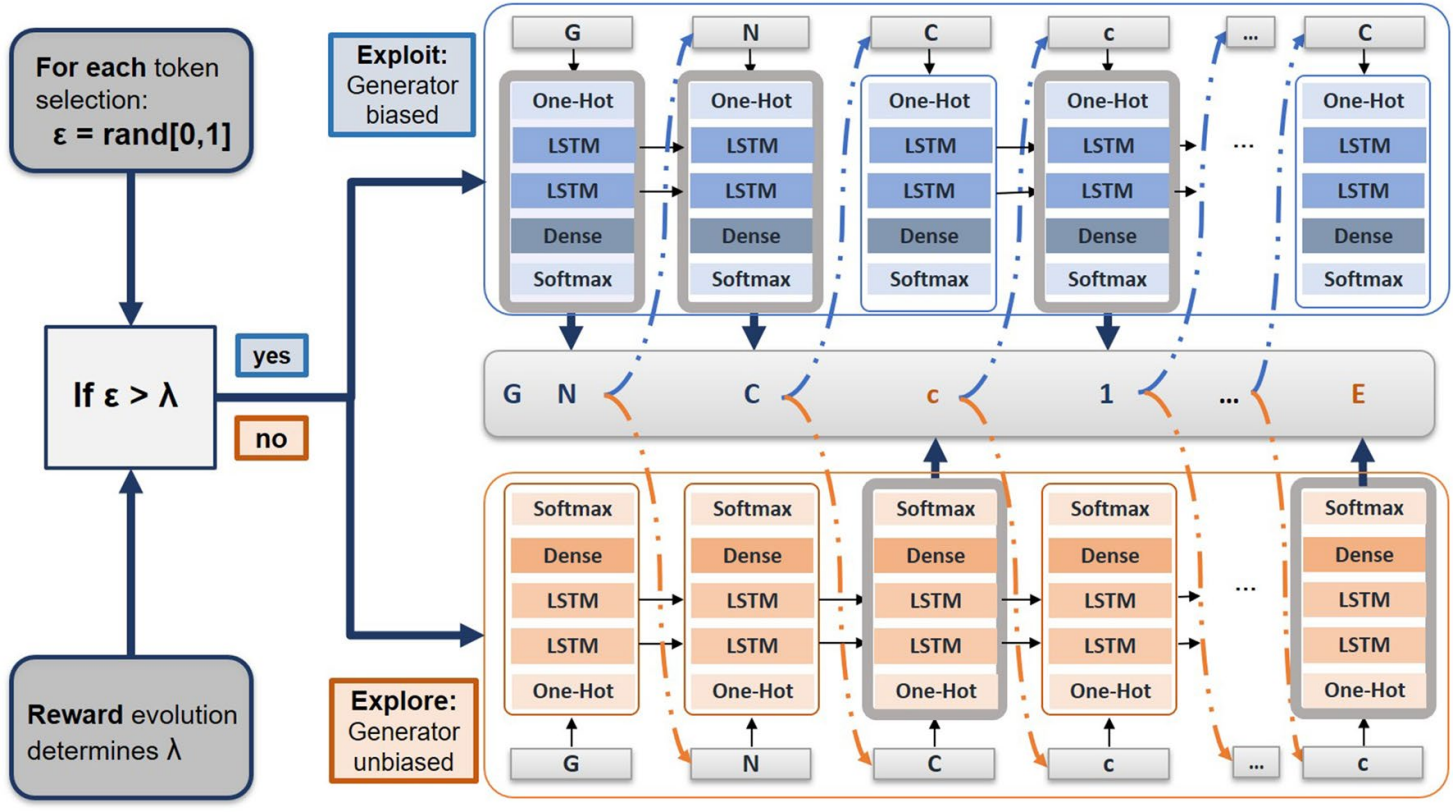
Process of generating SMILES using two Generators alternatively

The second way to handle this trade-off is through the alternate use of two policies to predict the next token. The innovation of this strategy relies on the fact that it takes into account not only the goal of discovering promising molecules but also the purpose of preserving the diversity of the compounds. In other words, the token selection in the generation of new SMILES strings is determined by two generators: the Generator biased, $G_{b}$, that has been updated by the RL approach, and a second fixed Generator, initially trained without RL, Generator unbiased, $G_{u}$. Since the SMILES are being constructed token by token and depending only on the part of the molecule hitherto built, it is possible to intercalate both Generators to predict the next token. The $G_{u}$ was only trained to generate synthesizable molecules, while the $G_{b}$ is being trained to maximize a cumulative reward. In this regard, the $G_{u}$ is associated with exploration, and the $G_{b}$ is responsible for exploitation. The frequency by which $G_{b}$ or $G_{u}$ are used is dynamically determined and depends on the evolution of the reward. Specifically, a random number $\epsilon$ between 0 and 1 is generated at each step. If the value is smaller than a threshold $\lambda$, the $G_{u}$ predicts the next token otherwise $G_{b}$ will be selected. Note that this threshold, $\lambda$, is dynamically determined as the RL process progresses. As a rule, if the averaged reward increases for the two last batches of generated molecules (the Generator is being updated in the right direction), the $\lambda$ decreases and exploitation is favoured. In contrast, if the reward is not improving as expected, the $\lambda$ increases in order to the $G_{u}$ policy be more often employed. The description of this process is depicted in Fig. 5.

Lastly, another procedure to preserve an appropriate novelty in the generated compounds is to reflect in the reward value a penalty when the novelty decreases. This adjustment in the reward is performed based on the analysis of the similarity between each created molecule and the collection of the last 30 generated molecules that will act as a continuously updated memory. The similarity between each molecule will be determined using Tanimoto distance, which will analyze the respective ECFP of the molecules to assign a value between 0 (molecules with identical fingerprints) and 1 (totally different fingerprints). Then, based on the calculated average diversity, the reward may suffer a penalty of 15% of its value if the reward is less than a threshold $\kappa$. In this regard, generating similar molecules consecutively will have negative repercussions on the reward value. Thereby, performing this correction, the weights will be adjusted in order to avoid the penalization, and the Generator will be able to get out of possible relative minimums.

### Evaluation metrics

It is essential for the de novo targeted lead generation to preserve the compounds’ validity, and their biological interest [10, 11]. Consequently, the obtained molecules should undergo a rigorous evaluation regarding their validity, desirability, synthetic accessibility, drug-likeness, uniqueness, and diversity. In this work, a molecule is defined as “valid” if it can pass through the RDKit sanitiser [29]. To generate molecules with a specific optimised property, a metric to assess is necessary. For the binding affinity the $\text{ pIC}_{50}$ is used and to define a compound as *desirable*, we set the threshold for greater than and equal to 6.5 similar to Liu et al. [17]. For the synthesizability evaluation, we applied a tool to assess the facility with which computationally designed compounds can be synthesised in practice. It is a metric named Synthetic Accessibility Score (SAS) that varies between 1 and 10. If a molecule has a score above 6, it is considered difficult to synthesise. Its estimation is based on the knowledge extracted from known synthetic reactions and penalisation of high molecular complexity [30].

We assess the diversity between the generated compounds based on three types of metrics. Tanimoto similarity ($T_s$), Levenshtein distance ($L_d$) and maximum common substructure (*MCS*) to ensure the robustness of the assessment. The objective is to take advantage of the complementary of the three metrics in order to draw valid conclusions regarding the diversity and novelty of the generated molecules.

$T_s$ ascertain the distance between molecular fingerprints. In this case, it is necessary to transform each SMILES into the respective ECFP3 format. This descriptor is a binary vector that is constructed so that the more similar two molecules are, the more elements are equal in the respective vectors. Based on this principle, $T_s$ between two molecules *a* and *b* is given by the following equation:

6 $$\begin{aligned} T_s(a,b) = \frac{|m_{a}|\cap |m_{b}|}{|m_{a}|\cup |m_{b}|} \end{aligned}$$

where $|m_{a}|\cap |m_{b}|$ indicate the coefficient between the number of matching bits and $|m_{a}|\cup |m_{b}|$ the total number of bits formed by the two compounds. Thus, $T_s$ vary between 0 and 1. The lower the result, the less similar the compounds are.

The second measure to evaluate molecular diversity is Levenshtein distance. It is a commonly employed parameter to measure the heterogeneity between two text strings [31]. As SMILES are essentially strings, Levenshtein distance can be applied directly on SMILES strings. This distance is defined as the minimal number of insertions, deletions, and replacements needed to make two SMILES identical. The equation 7 illustrates how the Levenshtein distance ($L_d$) is calculated between two SMILES strings of molecules *a* and *b*.

7 $$\begin{aligned} {\text {lev}}_{a, b}(i, j)=\left\{ \begin{array}{ll} \max (i, j) &{} \text{ if } \min (i, j)=0 \\ \min \left\{ \begin{array}{l} {\text {lev}}_{a, b}(i-1, j)+1 \\ {\text {lev}}_{a, b}(i, j-1)+1 \\ {\text {lev}}_{a, b}(i-1, j-1)+1_{\left( a_{i} \ne b_{j}\right) } \end{array}\right.&\text{ otherwise } \end{array}\right. \end{aligned}$$

In this equation, $1_{\left( a_{i} \ne b_{j}\right) }$ is an indicator function, which is equal to 1 if token (atom or bond) *i* of *a* is not equal to token *j* of *b* and 0 otherwise.

The last parameter to evaluate the molecular diversity is the *MCS* that is given by Eq. 8. This metric calculates the ratio between the number of atoms in the maximum common substructure of molecules $m_a$ and $m_b$ and the number of atoms of the molecules $m_a$ and $m_b$. The MCS-based approaches perform well in identifying local similarities where structural descriptor methods typically fail [32].

8 $$\begin{aligned} MCS\left( a, b\right) =\frac{{\text {mcs}}\left( m_{a}, m_{b}\right) }{ \text{ atoms } \left( m_{a}\right) + \text{ atoms } \left( m_{b}\right) -{\text {mcs}}\left( m_{a}, m_{b}\right) } \end{aligned}$$

Note that although $T_s$ and *MCS* indicate the similarity between compounds, it is possible to use these metrics for estimating the diversity through the calculation of $1-T_s$. Therefore, from these three definitions of distance, we calculate the diversity within a set of compounds (internal diversity) and the diversity between two sets of different molecules (external diversity). The basic principle is the same all metrics, i.e., we intend to compute the diversity between pairs of molecules and calculate the average values of all these comparisons. In the case of internal diversity, the sum of all diversities is divided by the square of the number of molecules in the set. In contrast, for external diversity, the division is made by the product of the molecules of the two sets. Equation 9 exemplifies the calculation of the external diversity between two sets of molecules *A* and *B*.

9 $$\begin{aligned} Div(A,B) = \frac{1}{|A|\cdot |B|} \sum _{a\in A}^{|A|} \sum _{b\in B}^{|B|} \left( distance(a,b)\right) \end{aligned}$$

where |*A*| and |*B*| represent the number of molecules in sets A and B, and *distance* is $1-T_s$, 1-MCS and $L_d$. In addition, for computing internal diversity, the only change in the expression 9 is to replace *B* with *A* [15]. Note that All code for calculating these evaluation metrics is available in the GitHub repository.

## Results

### Datasets

The Generator is trained with a set of 499,915 SMILES derived from the ZINC database (version 15) [33]. We randomly select molecules with a partition coefficient (logP) ranging from -2 to 6, and, molecular weight between 200 and 600g/mol. To train the Predictor networks, we extract SMILES strings, and the corresponding bioactivity against the targets from ChEMBL benchmark [34]. For the Adenosine $A_{2A}$ receptors $A_{2A}R$, 4,872 compounds are extracted (ChEMBL identifier: CHEMBL251) whereas for the KOR, we collect 7,102 compounds (ChEMBL identifier: CHEMBL3952). After performing the duplicates deletion, all SMILES strings are canonicalized using the RDKit tool [29]. Both receptors are types of G Protein-Coupled Receptors (GPCRs), and the $A_{2A}R$ is involved in the treatment of conditions such as insomnia, pain, depression, Parkinson’s disease, cardiovascular diseases, and inflammatory disorders [19], while the KOR mediates pain, mood, and motor control. The interest in KOR stems from its crucial role in pain control, and drug addiction issues [35].

### Experimental analysis on initial SMILES generator

After training the Generator, we sampled 10,000 new SMILES to evaluate its performance with respect to desirability, validity, diversity, synthesizability, and uniqueness. The results are summarized in Table 2. In this stage, the percentage of desirable molecules is not the priority since we are only interested in obtaining a model that learns the building rules of molecules through SMILES strings. According to RDKit parsing, nearly $91\%$ of the sampled molecules were chemically valid. Also, the internal and external Tanimoto diversities greater than 0.9, as well as the high rate of unique SMILES in the 10, 000 generated molecules, have demonstrated that the model can add variability and novelty compared to the dataset. The synthesizability of the molecules is highly likely since the obtained average SAS was 2.78. This value is well below 6, from which a molecule is considered difficult to synthesize. Hence, we can infer that the mentioned results are quite promising, i.e., they prove that recurrent architecture is an appropriate method to learn SMILES grammar. The unbiased Generator is the cornerstone of this work since it will be the starting point for all the subsequent experiments and must be reliable.

**Table 2:** Evaluation of unbiased generator

% Desirable	% Valid	Internal diversity	External diversity	SAS	% Unique
86.3	91.1	0.91	0.91	2.87	99.9

### QSAR models performance

**Fig. 6:**
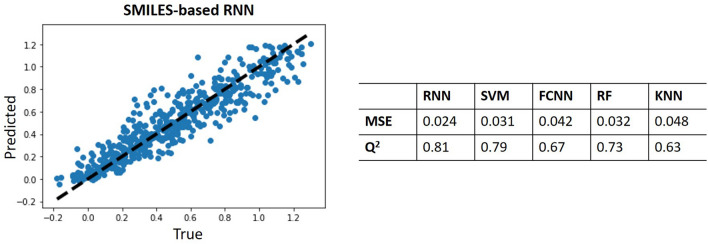
Performance of different QSAR approaches: scatter diagram and analysis of MSE and *Q*^2^

Two different strategies are implemented to obtain the QSAR model that established the mapping between the newly generated molecules and its affinity for the target. We aimed to verify that it is possible to exceed the performance of the models that used the traditional ECFP vectors as a molecular descriptor by employing an apparently more rudimentary and straightforward approach to obtain a descriptor such as SMILES. Different algorithms for the QSAR implementation are evaluated by computing regression-like metrics such as the Mean Squared Error (MSE) and $Q^2$ [36]. Figure 6 summarizes the obtained results for the $A_{2A}R$ Predictor.

From the analysis of both metrics, it is noticeable that the SMILES-based QSAR provides more reliable information regarding the biological affinity of new compounds. This strategy outperforms the traditional ECFP-based methods (both the standard approaches and the FCNN), demonstrating that SMILES notation contains valuable embedded information about the compounds for the construction of QSARs and thereby, it will be used in the subsequent experiments.

### Analysis of biased SMILES generator

We implement a grid search strategy to handle certain hyper-parameters that ensure the RL method’s proper behaviour while minimizing the loss function. The approach is repeated for 85 iterations, using Adam optimizer with 0.001 as the learning rate. Moreover, each batch contained ten molecules, the Softmax temperature is set to 0.9, and gradients clipped to [−3, 3]. Finally, the conversion from the predicted $\text{ pIC}_{50}$ of the molecule to the assigned reward is performed using the following rule: $R_{t}$ = $exp(\frac{pIC_{50}}{4} - 1)$. It should be mentioned that Additional file 1 contains the necessary information to ensure the reproducibility of each module of this framework in its entirety with the optimal parameters.

#### Optimizing molecular properties

**Fig. 7:**
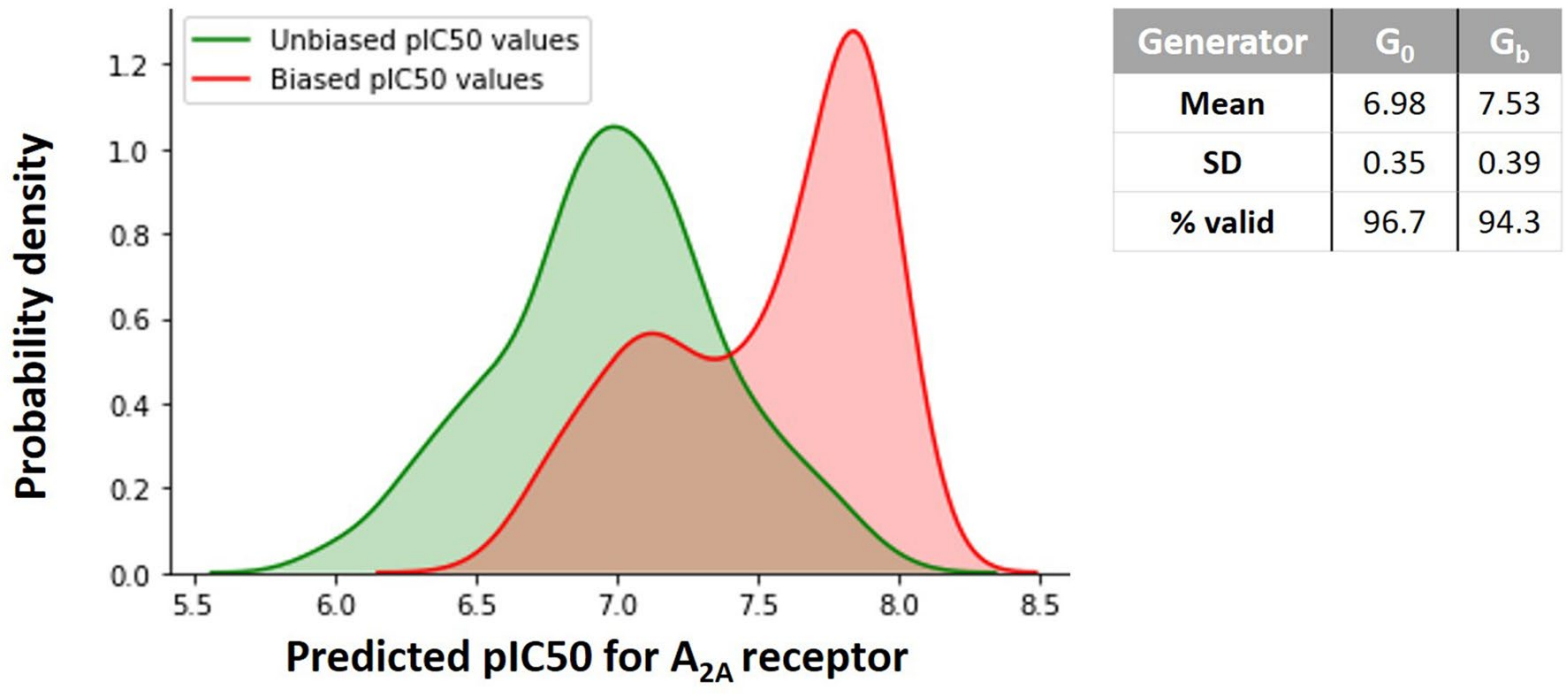
Comparison of pIC_50_ distributions for $A_{2A}R$ before and after applying Reinforcement Learning

In the first proof-of-concept experiment, we aimed to generate molecules that are likely to inhibit $A_{2A}R$ antagonistically by maximizing the $\text{ pIC}_{50}$ of generated compounds. Figure 7 represents the biasing of the Generator instilled by the application of RL. It compares the probability density of the predicted $\text{ pIC}_{50}$ for $A_{2A}R$ obtained from the unbiased Generator and after the RL training process. It is noticeable that after retraining the Generator with RL, the likelihood of generating molecules with a higher $\text{ pIC}_{50}$ increases, and the validity rate remains nearly the same for both distributions. Therefore, the newly created molecules have a more significant potential to inhibit the receptor mentioned above.

**Fig. 8:**
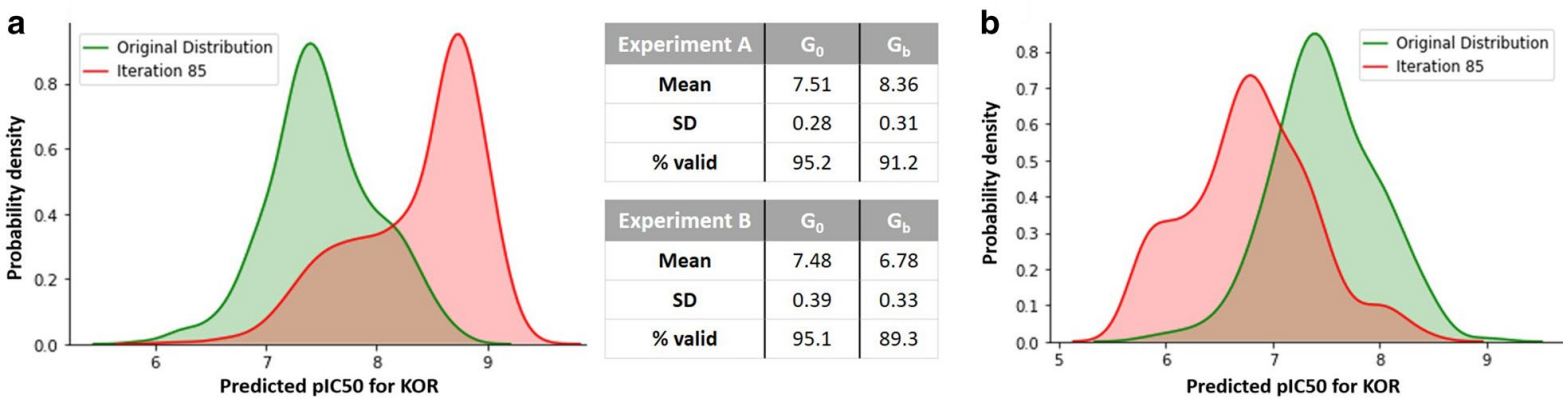
Comparison of pIC_50_ distributions for KOR before and after applying RL. **a** Maximization of biological affinity. **b** Minimization of biological affinity

Afterwards, the same procedures are applied for a different receptor to demonstrate the versatility of this framework. Hence, KOR is used as a target for the new compounds, and we exam the training of the corresponding Predictor. However, in this case, in addition to the experiment that aimed to maximize the affinity towards the receptor, another experiment is carried out with the opposite objective of minimizing the $\text{ pIC}_{50}$. In the latter, the Generator weights are updated so that it would favour the generation of compounds having a low affinity for the KOR. This type of optimization can avoid the off-target effects since it can reduce the affinity of a potential drug to known competing targets. The result of applying the RL is shown in Fig. 8 that shows the distributions are correctly skewed in both cases, and the percentage of chemically valid molecules is kept.

**Fig. 9:**
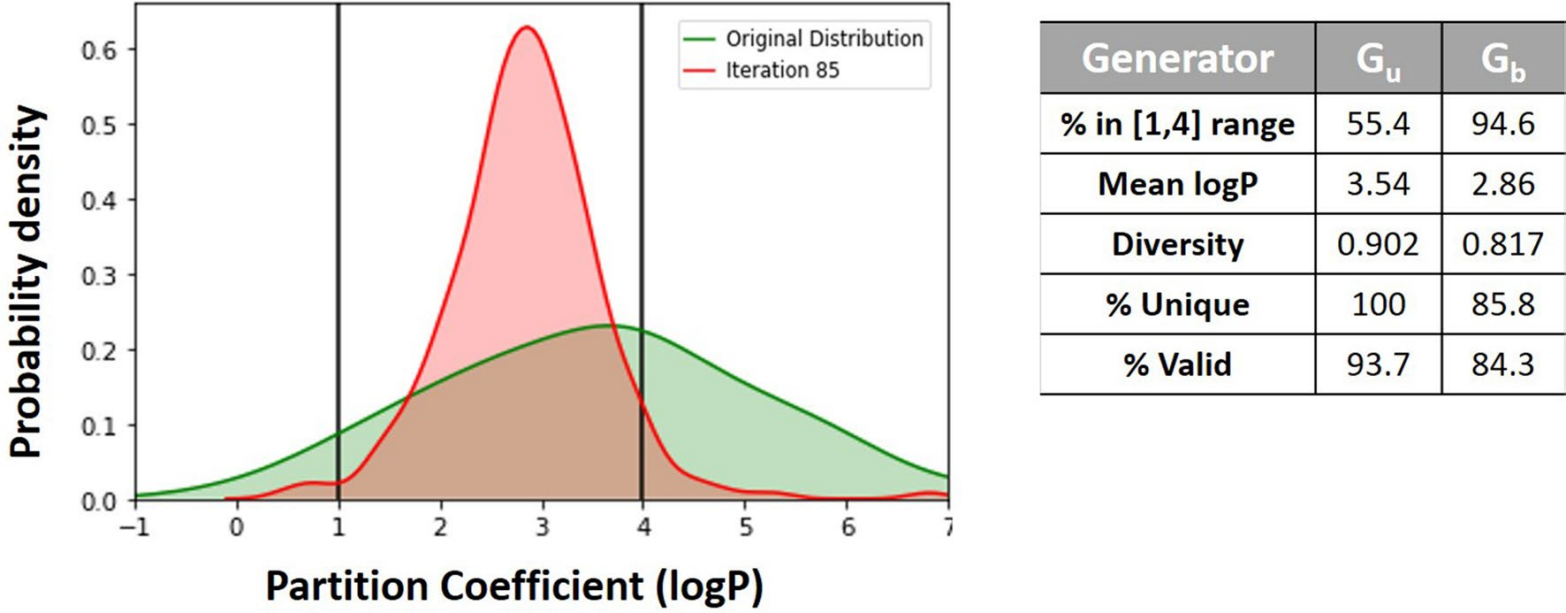
Comparison of logP distributions and molecular properties before and after applying RL

The third experiment analysis aims to confirm this methodology’s versatility and prove the ability to bias the property of interest and maintain the diversity independent of the Predicting property. We perform the optimisation for the partition coefficient logarithm (logP) property. This molecular property does not require a Predictor model as it is extensively studied in the literature and RDKit toolkit can apply for the calculation. The logP value provides indications of the lipophilicity of the compounds. Consequently, it is a crucial property for the compounds’ permeability across the body’s membranes composed of a phospholipid bilayer. For a compound to have necessary features in terms of bioavailability, it is essential to have a logP in a range between 1 and 4, according to the Lipinski’s rule of five [11]. In this experiment, we intended to fine-tune the Generator to generate molecules with logP within this range. Figure 9 summarises the comparison of logP distributions before and after application of RL.

There is an evident change in the logP distribution comparing the molecules generated before and after the application of RL. Moreover, it demonstrates that biasing of properties and maintenance of the diversity in the molecules occurs regardless of whether the property needs a model for predicting on-target activity or not.

#### Novelty evaluation—comparison with other methods

The developed strategy to ensure greater diversity in the generated compounds includes the manipulation of the reward value. It is assigned a penalty to the model when it starts to output compounds with reduced diversity. The search space for the diversity threshold ($\kappa$) below which the model is penalized is [0.7, 0.75]. On the other hand, the use of two generators alternately, depending on the evolution of the assigned reward, is a straightforward way to conduct the search process through promising chemical spaces and, at the same time, to keep the novelty in the resulting compounds. A threshold ($\lambda$) from a set of user-defined thresholds are applied to determine the Generator selected to predict the next token more often. When the averaged reward of the previous batches of molecules increases, the smaller $\lambda$ is applied. Alternatively, if the averaged reward is decreasing, it is selected the higher $\lambda$ and, if the reward does not show a defined trend, an intermediate value for $\lambda$ is selected. The set of thresholds are called $\tau$ and the verified alternatives are [0,0,0] ($\tau _{1}$) to work as a baseline, [0.05,0.2,0.1] ($\tau _{2}$), [0.1,0.25,0.15] ($\tau _{3}$) and [0.15,0.3,0.2] ($\tau _{4}$). The baseline approach is implemented without both strategies that control the Generator’s exploratory behaviour to observe their influence on the properties of the obtained molecules. The evaluation metrics are computed after the generation of 10,000 molecules. Table 3 outlines the results when testing the possible combinations of the described parameters. The diversity metric is the average Tanimoto distance between the pairs of molecules in the set under evaluation. The search spaces for both strategies to deal with the exploration/exploitation dilemma are empirically established to privilege the compounds’ diversity while preserving the model’s ability to shift the molecules’ property in the desired direction.

**Table 3:** The obtained results employing different configurations of the parameters that affect the exploratory behaviour of the model

Results							
$\tau$	1	2	2	3	3	4	4
$\kappa$	-	0.70	0.75	0.70	0.75	0.70	0.75
% Desirable	99.8	91.1	95.1	98.1	92.9	96.3	95.8
% Unique	10.3	72.3	96.4	53.5	79.2	83.3	89.2
% Validity	97.4	88.7	90.4	82.8	77.1	74.3	82.2
Diversity	0.768	0.827	0.879	0.851	0.857	0.887	0.891

The first conclusion that can be extracted from all experiments is the increase in the percentage of desirable molecules compared to the previously trained Generator without RL (Table 2). In addition, the rate of chemically valid molecules has slightly decreased. However, it is possible to identify specific configurations in which this percentage remained close to 90%, which demonstrates the robustness of the method. As previously mentioned, the novelty of the newly generated molecules was measured with two parameters: the average internal Tanimoto distance between pairs of molecules and by computing the percentage of unique molecules. These indicators are intended to ascertain whether the policy falls into a relative minimum of the loss that only reproduces very similar molecules or if it manages to generate a set of lead compounds with interesting diversity. Table 3 shows specific parameter configurations that produce valuable Generators, i.e., models with uniqueness, diversity and desirability rates that correspond to policies with the potential to generate lead compounds. Moreover, as the selected $\tau$ favours more the exploratory behaviour of the model (from $\tau _{1}$ to $\tau _{4}$), it is visible an increase in the diversity and percentage of unique molecules. In opposition, the desirability tends to decrease when one chooses to guarantee more significant novelty. As far as the parameters that influence the penalty of the model are concerned, we see that when the reward penalty occurs for similarities less than 0.75, the percentage of unique molecules is higher, and the diversity slightly increases when compared with the 0.7 $\alpha$. This is a foreseen evidence since by penalizing the similarity more strongly (from 0.75 Tanimoto distance), the Generator will obtain greater diversity in the molecules at the end of the training process.

The adjustment of parameters that ensured the most appropriate compromise between the desired properties occurred when $\tau _{2}$ was applied, and 0.75 was defined for the $\kappa$ threshold. It should be noted that the best policy was not obtained at the end of the training process, but at an earlier stage, when the compromise between diversity and desirability was more favourable. Therefore, it was possible to achieve a high percentage of molecules having a $\text{ pIC}_ {50}$ higher than 6.5, preserving the validity rate. Moreover, our exploratory strategies have the wanted consequence, as the average Tanimoto diversity significantly increased when compared to the other approaches, as shown in Table 4. The results achieved by REINVENT, ORGANIC and DrugEx were collected from the work of Liu et al. regarding the $A_{2A}R$. The models were executed using their default parameters. Then, 10,000 molecules were generated and computed the average Tanimoto distance between all the pairs of compounds. Table 4 shows that those works have percentages of chemically valid higher than our approach. In terms of desirability and uniqueness, our work managed to reach comparable results. However, diversity has increased considerably compared to all studies, which demonstrates the efficacy of this solution.

**Table 4:** Comparison of our approach with other generative methods

	DrugEx	REINVENT	ORGANIC	Best configuration
${\varvec{\tau }}$	–	–	–	2
${\varvec{\alpha }}$	–	–	–	0.7
${\varvec{\lambda }}$	–	–	–	0.9
% Desirable	98.5	98.2	99.8	95.1
% Unique	99.1	95.8	94.8	92.9
% Validity	99.0	98.8	99.8	90.4
Diversity	0.74	0.75	0.67	0.86

The diversity analysis has been extended to demonstrate our framework’s capacity to maintain the diversity of compounds as the training process takes place. This analysis now includes two additional metrics besides the Tanimoto diversity: Levenshtein distance and MCS. Table 5 indicates the obtained values of each metric for the unbiased Generator (training step 0) and biased Generator (training steps 150, 300 and 450).

**Table 5:** Evaluation of molecular diversity throughout the training process applying three distance measure

Training step	0	150	300	450
Tanimoto distance	0.901	0.877	0.857	0.824
Levenshtein distance	31.2	26.8	24.3	22.1
MCS distance	0.799	0.767	0.724	0.713

As discussed in “Evaluation metrics” section, these three metrics have very different ways of approaching the molecular diversity estimation. Thus, we aim to take advantage of these metrics’ complementarity to generalize the assessment of diversity and demonstrate that the conclusions drawn are meaningful, regardless of the assessment parameter. Table 5 indicates a slight decrease in molecular diversity as the training process progresses that is noticeable for the three metrics. Also, it should be emphasized that even for the biased Generator (step 450), diversity is maintained at a satisfactory level, i.e., higher than the values observed in other works.

## Discussion

The performed experiments have shown a trade-off between how skewed the property of interest is and the novelty associated with the generated molecules. The more the desired property is shifted in the right direction, the more the diversity decreased, with a noticeable rise in the number of repeated molecules. The reason for this decrease in diversity is that, as the weights of the Generator are updated, the loss function may reach possible minimums that although they correspond to the generation of molecules with high reward attributed by the Predictor, they also cause a significant reduction in the diversity of compounds.

However, the trade-off between desirability and diversity is complex to handle. Despite the fact the Generator’s main goal is to produce molecules with the correctly biased properties, it is no less critical to keeping the variability. This trade-off, which ultimately reveals itself in the molecule’s characteristics, is originated from the exploration/exploitation dilemma. From the analysis of the evolution of all experiments, it is clear that, when the Generator is giving more importance to exploitation, one of two unwanted things can happen. On the one hand, the policy can “fall” into a local minimum, with no interest in the problem context, which continues to exploit the reward function fruitlessly. For instance, the model can produce SMILES strings that are sequences of merely carbon atoms, without any biological significance. On the other hand, the policy’s continuous updates can lead the Generator to a minimum, which, having a significant interest in the problem, substantially reduces the variability of the molecules produced by the Generator. Hence, the main challenge to tackle is how to deal with the exploration/exploitation dilemma. To find the balance between not having sufficient exploitative behaviour for the model to converge in the right direction and having excessive exploitation that causes the model to fall into one of the unwanted situations described above, it is necessary a precise adjustment of the parameters. The baseline approach allowed us to understand the importance of this accurate balance. In this case, only the updated Generator is being used to predict the next token during the training process and, as a result, only exploitation is being taken into account. Moreover, the strategy of using thresholds to minimize the repetitive generation of molecules has not been used in this configuration. Therefore, the results demonstrate a remarkable decrease in the novelty of the generated molecules, which confirms the usefulness of these strategies. This finding is corroborated by the best Generator in which the trade-off between desirability and novelty is correctly established.

**Fig. 10:**
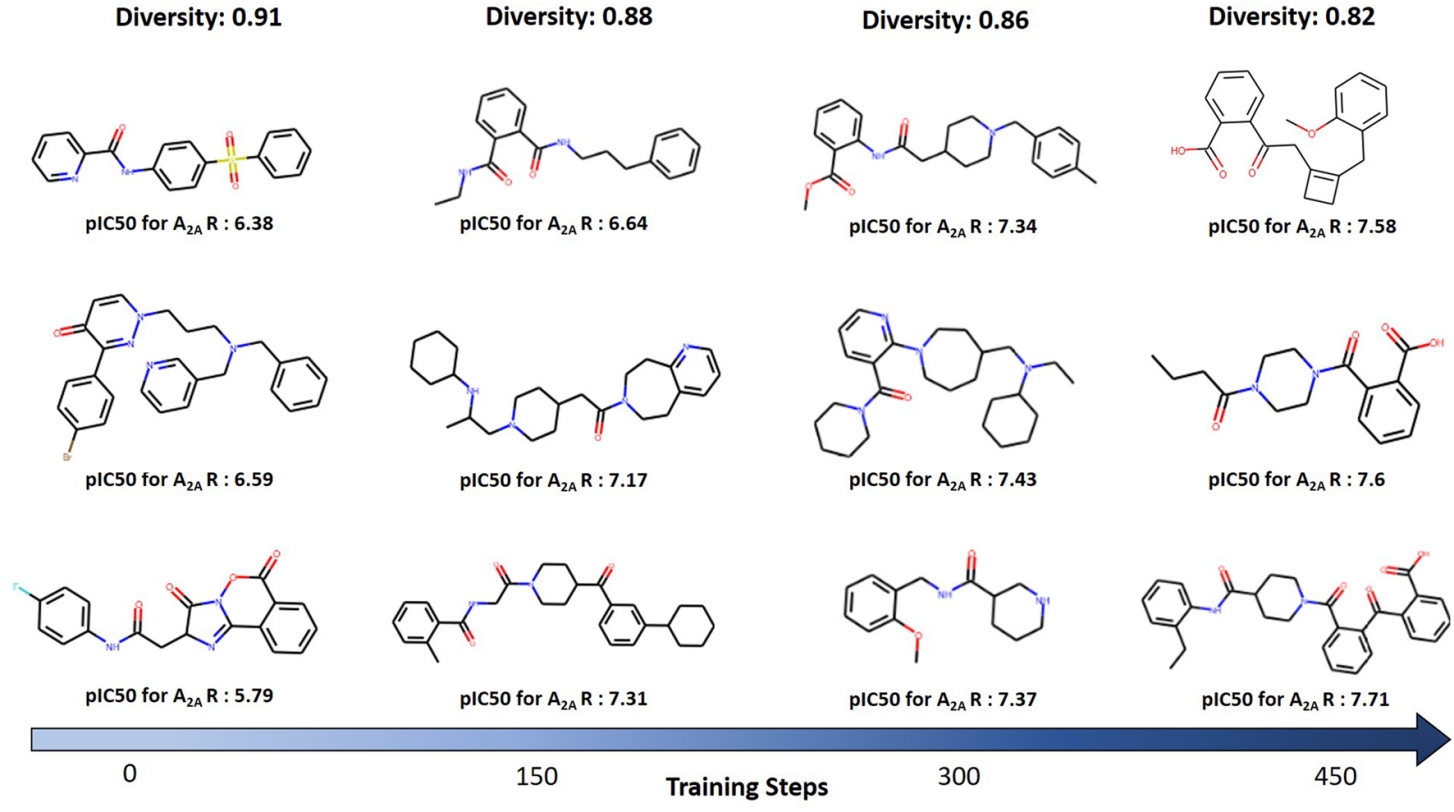
Examples of molecules obtained at different stages of the training process illustrating the tradeoff between diversity and biological affinity for the target

Figure 10 exemplifies molecules produced by the biased Generator at different stages of the training process. At each step, we generate 500 molecules, and the corresponding average Tanimoto distance for that set is placed at the top. Also, we represent the respective affinity for $A_{2A}R$ for each molecule. As training steps increase, there is a tendency to increase the estimated inhibition power of the molecules and to slightly decrease average diversity, even though it always remains at a satisfactory level.

## Conclusions

In this work, we propose a molecule generation framework that combines SMILES-based models implemented with recurrent architectures and RL for the targeted generation of molecules. Both the Generator and the Predictor are initially trained with supervised learning. The purpose of the former is to learn the rules for building valid molecules. For the latter, the goal is to predict the biological affinity of molecules for the target of interest. Both models have demonstrated the ability of this type of architecture to operate with SMILES notation.

We also extend other works that have already used RL to generate molecules with optimized properties. Thus, by integrating the REINFORCE method with exploratory strategies implemented during the training process, it is possible to obtain a set of active molecules for the desired target and to get more diversity than in other comparable methods. In this framework, the Predictor acted as an evaluator of the newly created molecules and contributed to give them a reward based on the goodness of the desired property. On that account, we have shown that the model employing SMILES strings as input data is more robust predicting biological properties than the alternative models such as FCNN, SVR, RF, and KNN based on the standard descriptor (ECFP).

Throughout this work, the model’s versatility has been demonstrated by changing the properties distribution of the molecules in different contexts by maximizing a reward function. In addition, with our exploratory strategies, it is possible to bias the generation process towards the goal and preserve the novelty and validity of the compounds at quite reasonable levels. Furthermore, the comparison with other methods allowed to confirm the effectiveness of the implemented strategy since it was possible to obtain molecules having more Tanimoto diversity and a similar percentage of desirability. We demonstrate the importance of the exploration/exploitation dilemma in RL to avoid the fruitless exploitation of the reward function. Nevertheless, although there is room to improve the model, the implemented framework for de novo lead generation is another step forward towards a more prominent application of computational methods in the drug discovery process.

## Future work

After the creation of this framework for the targeted generation of molecules, one of the obvious next steps is the extension of this method to optimize more than one property at the same time. In addition to biological properties, the next version of this work will also consider the physical and chemical features of the generated molecules. This improvement could integrate RL with multi-objective optimization and thereby bring the complexity of the method closer to the demanding standards required in the development of new candidate drugs.

## Supplementary Information

**Additional file 1.** The supplementary file contains all the required dependencies to ensure compatibility with the provided code. Furthermore, all the necessary steps for implementing each module of the framework are indicated in detail.

## Data Availability

The datasets analysed during the current study are available in the ZINC (http://zinc15.docking.org/) and CHEmbl (https://www.ebi.ac.uk/chembl/) repositories. All code is publicly available in the https://github.com/larngroup/DiverseDRL
