# Supplementary material for: Diversity oriented Deep Reinforcement Learning for targeted molecule generation
Source: J Cheminform. 2021 Mar 9;13:21. doi: 10.1186/s13321-021-00498-z (PMC7944916; doi:10.1186/s13321-021-00498-z)
Supplement: Supplementary file 1 — Additional file 1. The supplementary file contains all the required dependencies to ensure compatibility with the provided code. Furthermore, all the necessary steps for implementing each module of the framework are indicated in detail. [file 13321_2021_498_MOESM1_ESM.pdf]

Introduction

In this work, we explore the potential of deep learning to streamline the process of identifying new potential drugs through the computational generation of molecules with interesting biological properties. Two deep neural networks compose our targeted generation framework: the Generator, which is trained to learn the building rules of valid molecules employing SMILES strings notation, and the Predictor which evaluates the newly generated compounds by predicting their affinity for the desired target. Then, the Generator is optimised through Reinforcement Learning to produce molecules with bespoken properties.

The innovation of this approach is the exploratory strategy applied during the reinforcement training process that seeks to add novelty to the generated compounds. This training strategy employs two Generators interchangeably to sample new SMILES: the initially trained model that will remain fixed and a copy of the previous one that will be updated during the training to uncover the most promising molecules. The evolution of the reward assigned by the Predictor determines how often each one is employed to select the next token of the molecule. This strategy establishes a compromise between the need to acquire more information about the chemical space and the need to sample new molecules, with the experience gained so far.

To demonstrate the effectiveness of the method, the Generator is trained to design molecules with an optimised coefficient of partition and high inhibitory power against the adenosine A2A and k-opioid receptors. The results reveal that the model can effectively adjust the newly generated molecules towards the wanted direction. More importantly, it is possible to find promising sets of unique and diverse molecules, which is the main purpose of the newly implemented strategy.

Workflow

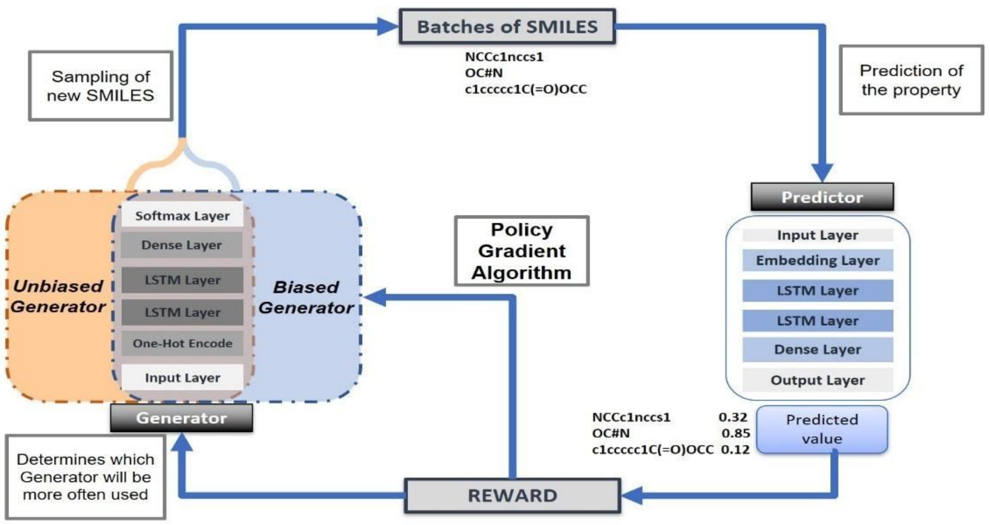

Exploratory Strategy

- Softmax activation function.
- Two Generator methodology based on the evolution of the reward.

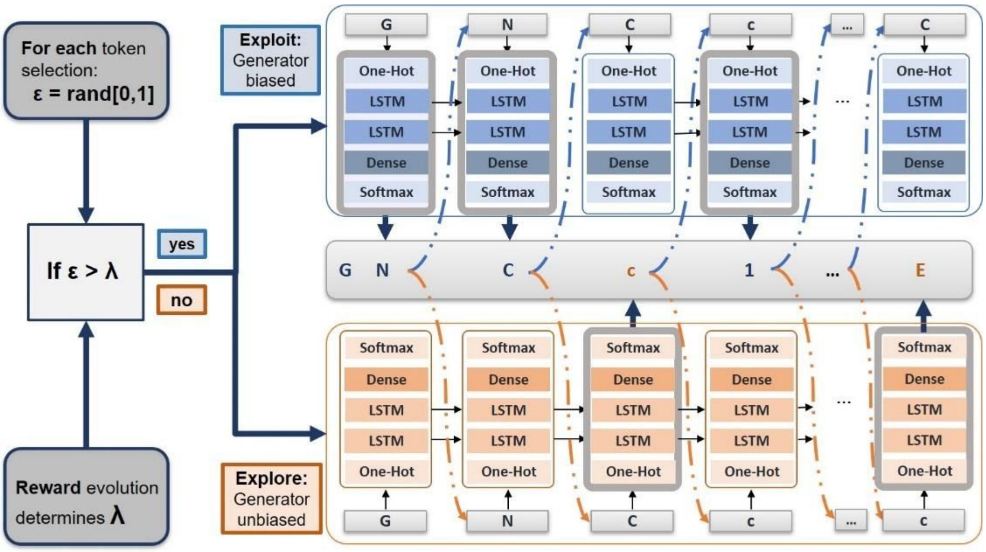

- Memory regularizer to penalize repetitive generation

Dependencies

All the code is implemented on Python. The Python version should be greater and equal to 3.6. The following packages are necessary to install:

- Scikit-Learning (version >= 0.23)
    - pip install scikit-learn
- Numpy (version >= 1.17)
    - pip install numpy
- Seaborn (version >= 0.9)
    - pip install seaborn
- Tqdm (version >= 4.37)
    - pip install tqdm
- Tensorflow (version >= 1.13)
    - Pip install tensorflow
- Keras (version >= 2.3.1)
    - pip install Keras
- Bunch (version >= 1.0)
    - pip install bunch
- Json (version >= 3.0)
    - pip install jsonschema
- RDKit (version >= 2019.09)
    - conda install -c conda-forge rdkit
- Pandas (version >= 0.25)
    - pip install pandas
- Matplotlib (version >= 3.1.1)
    - pip install matplotlib

Usage

The reproducibility of all modules that form the framework (the unbiased Generator, the Predictor, and the Generator's training process through Reinforcement Learning) is completely ensured. The unbiased Generator and the necessary Predictors were previously trained and ready to be employed. These models are placed in the folders 'generator model', 'predictor_models_a2d' and 'predictor_models_kor, respectively. However, they can also be obtained directly by running the respective codes present in the 'SmileGenerator' and 'Predictor' folders:

- **Unbiased Generator:** Access the SmileGenerator folder and execute the main.py file. Place the obtained files in the 'generator_model' folder.

- **Predictor:** Access the Predictor folder and execute the main.py file. There are several hypotheses for the Predictor architecture and, as such, it is necessary to choose whether to implement the Predictor based on deep-learning ('dnn') or machine learning ('SVR', 'RF', or 'KNN') and whether the descriptor to employ is 'SMILES' notation or 'ECFP'. Place the obtained files in the 'predictor_models_a2d' folder.

- **Biased Generator:** Execute the 'mainReinforce.py' file. It is necessary to specify a priori what is the objective to optimize: the biological affinity for Adenosine A2A ('a2a'), k-opioid receptor (kor), or partition coefficient (logP). Thus, the unbiased Generator and the respective Predictor models are loaded. Regardless of the objective, first, a set of molecules is generated from the initial Generator, through which Table 2 in the Results section is constructed. Then, Reinforcement Learning is applied to obtain the biased Generator. Finally, another set of molecules is generated to be compared with the initially generated set.

It must be emphasized that all the parameters can be adjusted by the user in the 'configReinforce.json' file. The default setting is the one that guaranteed the results depicted in the paper.
